# Comparison of the Pull‐Out Strength between a Novel Micro‐Dynamic Pedicle Screw and a Traditional Pedicle Screw in Lumbar Spine

**DOI:** 10.1111/os.12742

**Published:** 2020-08-09

**Authors:** Lei Qian, Weidong Chen, Peng Li, Dongbin Qu, Wenjie Liang, Minghui Zheng, Jun Ouyang

**Affiliations:** ^1^ Department of Anatomy, Southern Medical University Guangdong Provincial Key laboratory of Medical Biomechanics Shenzhen Digital Orthopedic Engineering Laboratory Guangzhou China; ^2^ Department of Spinal Surgery Nanfang Hospital, Southern Medical University Guangzhou China; ^3^ Department of Orthopedics, The Third Affiliated Hospital Southern Medical University, Guangdong Provincial Key Laboratory of Bone and Joint Degeneration Diseases Guangzhou China

**Keywords:** Micro‐dynamic, Pedicle crew, Pull‐out strength, Screw loosening, Spinal fusion

## Abstract

**Objective:**

This study aimed to investigate the strength of a novel micro‐dynamic pedicle screw by comparing it to the traditional pedicle screw.

**Methods:**

Forty‐five lumbar vertebrae received a traditional pedicle screw on one side and a micro‐dynamic pedicle screw on the other side as follows (traditional group *vs* micro‐dynamic group): 15 vertebrae underwent instant pull‐out testing; 15 vertebrae underwent 5000‐cyclic fatigue loading testing; and 15 vertebrae underwent 10,000‐cyclic fatigue loading testing and micro‐computed tomography (micro‐CT) scanning. The peek pull‐out force and normalized peek pull‐out force after instant pull‐out testing, 5000‐cyclic and 10,000‐cyclic fatigue loading testing were recorded to estimate the resistance of two types of screws. Bone mineral density was recorded to investigate the strength of the different screws in osteoporotic patients. And the semidiameter of the screw insertion area on micro‐CT images after fatigue were compared to describe the performance between screw and bone surface.

**Results:**

The bone mineral density showed a weak correlation with peek pull‐out force (r = 0.252, *P* = 0.024). The peek pull‐out force of traditional pedicle screw after 10,000‐cyclic fatigue loading were smaller than that of instant pull‐out test in both osteoporotic (*P* = 0.017) and healthy group (*P* = 0.029), the peek pull‐out force of micro‐dynamic pedicle screw after 10,000‐cyclic fatigue loading was smaller than that in instant pull‐out test in osteoporotic group (*P* = 0.033), but no significant difference in healthy group (*P* = 0.853). The peek pull‐out force in traditional group and micro‐dynamic group underwent instant pull‐out testing (*P* = 0.485), and pull‐out testing after 5000‐cyclic fatigue loading testing (*P* = 0.184) did not show significant difference. However, the peek pull‐out force in micro‐dynamic group underwent pull‐test after 10,000‐cyclic fatigue loading testing was significantly greater than that measured in traditional group (*P* = 0.005). The normalized peek pull‐out force of traditional groups underwent instant pull‐out testing, pull‐out test after 5000‐cyclic and 10,000‐cyclic fatigue loading testing significantly decreased as the number of cycles increased (*P* < 0.001); meanwhile, the normalized peek pull‐out force of micro‐dynamic groups remained consistent regardless of the number of cycles (*P* = 0.133). The semidiameter after the fatigue loading test of the traditional screw insertion area was significantly larger than that of the micro‐dynamic screw insertion area (*P* = 0.013).

**Conclusion:**

The novel micro‐dynamic pedicle screw provides stronger fixation stability in high‐cyclic fatigue loading and non‐osteoporotic patients *versus* the traditional pedicle screw, but similar resistance in low‐cycle fatigue testing and osteoporotic group vs the traditional pedicle screw.

## Introduction

Spinal fusion and rigid fixation have been the main surgical treatment options for spine surgery, such as lumbar degenerative disease, lumbar instability and scoliosis^1^, ^2^. Although rigid fixation can provide great biomechanical stability, potential complications associated with this approach have been reported in recent years. The prevention of motion of the surgical segment leads to high stress on the fixation implant, increasing mobility, and intradiscal pressure at adjacent levels^3^. This may result in adjacent segment degeneration and loosening/rupture of the fixation implant^4^, ^5^, ^6^, ^7^, ^8^, ^9^. Non‐fusion and dynamic fusion instruments have been designed to prevent adjacent segment disease. Among those dynamic fusion instruments, the posterior dynamic stabilization devices are most commonly used. The posterior pedicle screw‐based flexible devices aim to allow micro motion between segment and also provide stability to reduce the back pain. The other dynamic stabilization devices such as Interspinous Process Distraction devices can decompress the intervertebral disc and nerve by expanding the space between interspinous, without pedicle screw insertion^10^. However, for devices such as the X‐Stop (St. Francis Medical Technologies, Alameda, California) and DIAM (Medtronic Sofamor Danek, Memphis,Tennessee) used in minimally invasive spine surgery, spinous process fracture may be difficult to avoid^11^, ^12^.

The pedicle screw based posterior dynamic stabilization devices are frequently used as dynamic fusion devices now. Numerous pedicle‐based dynamic fixation systems are currently available in an attempt to overcome the limitation of rigid fixation^13^, ^14^. The characteristic of those pedicle‐based dynamic fixation systems is the moving design to prevent degeneration of adjacent segments. Among those dynamic fusion systems, several systems provide pivoting of the screw head with little longitudinal translation, such as the Dynesys and the Cosmic (Ulrich Medical GmbH, Ulm, Germany). Others provide longitudinal translation with limited pivoting of the head, such as the DSS Stabilization System (Paradigm Spine, LLC, New York, NY, USA) and the N‐Flex (Synthes Spine Co., West Chester, MA, USA). Among these designs, the Dynesys and Cosmic systems are the most widely used with a larger body of evidence available from follow‐up studies. A literature review on Dynesys system conducted by the National Institute for Clinical Excellence of the National Health Service in the United Kingdom has shown that the Dynesys system can be helpful for patients with intractable lumbar pain^15^. Nevertheless, studies have reported that patients receiving the Dynesys system complain of both back and leg pain, with a relatively high infection rate^16^. A 2‐year follow up study reported that Cosmic system can provide safe stabilization and release the pain for the patients with disc herniation^17^. However, the hinge joint of the Cosmic system does not prevent stress concentration, leading to damage of the screw.

**Fig. 1:**
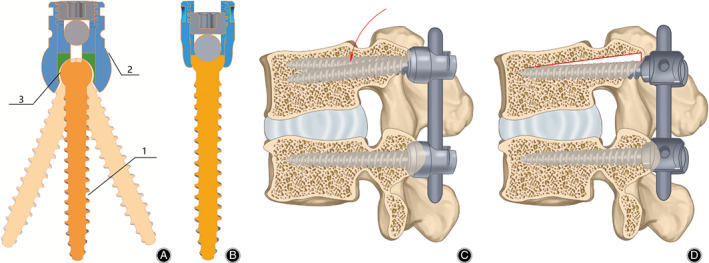
(A) The novel screw includes the nail body (1) and tail (2) and the ball‐and‐socket joint (3) connects the nail body and tail, the ball‐and‐socket joint can provide movement between the nail and tail within 6°, which provide micro motion between vertebral body and fixation instruments; (B) The traditional pedicle screw, to maintain consistent design of novel micro‐dynamic pedicle screw and traditional pedicle screw is same, the resultant thread length and diameter of the micro‐dynamic pedicle screw is same to that of the traditional screw; (C) The connection between the body and nail in micro‐dynamic pedicle screw can provide movement following the spinal motion (red arrow) through a ball‐and‐socket joint and prevent instability without screw loosening; (D) As the rigid fixation between traditional pedicle screw and vertebral body, the screw cannot move following the spinal motion, thus, screw loosening (blank space with red border) might happen after long time spinal movement.

The current understanding of the dynamic spinal fusion instrument is to preserve micro motion as well as provide safe stabilization. But it is difficult for most dynamic devices to provide the same longtime effective stabilization as the non‐rigid design. Thus, a micro‐dynamic design based on pedicle screw fusion system is needed. Several studies about dynamic pedicle screw fusion system have been reported, and the dynamic pedicle screw fusion system is such a design, aiming to reduce the concentration of stress on implants and preserve the motion of instrumented and adjacent segments^18^, ^19^. Our team have designed such a novel micro‐dynamic pedicle screw. The connection between the bone and the nail was achieved using a ball‐and‐socket joint, allowing the nail to move within this structure. And the ball‐and‐socket joint restricts the motion between the nail and tail within 6°, to preserve the micro‐motion locking of the screw‐rod joint and prevent excessive motion which may lead to failure of the fusion. The novel micro‐dynamic pedicle screw is composed of two parts (i.e., the nail body and nail tail). The connection between the body and nail is achieved through a ball‐and‐socket joint that can provide motion in all planes. Besides, the design of the ball‐and‐socket joint restricts motion between the nail and tail within 6°, to achieve reasonable physiological motion and prevent instability caused by large motion. The thread length and diameter of the novel pedicle screw is identical to that of the traditional pedicle screw (Fig. 1).

Recent studies indicated that the micro‐dynamic pedicle screw design provides an improved range of motion and load transfer behavior vs conventional pedicle screws^18^, ^20^. However, the resistance performance of the micro‐dynamic pedicle screw, especially in the situation of fatigue loading, remains unknown.

The purpose of this study was to investigate the effectiveness of the novel micro‐dynamic pedicle screw for the prevention of loosening after instant insertion and fatigue loading, the resistance of the novel micro‐dynamic pedicle screw in osteoporotic patients, and the difference in the performance between the novel micro‐dynamic pedicle screw and traditional pedicle screw between the screw and bone surface in lumbar spinal fusion. For this evaluation, the pull‐out force after fatigue resistance testing at different cycles was compared between the micro‐dynamic screw and the traditional pedicle screw. In addition, the fixation strength of two types of screws was examined by comparing the surrounding bone structure of implants on micro‐computed tomography (micro‐CT) images.

## Materials and Methods

### *Ethical Approval*

The experimental design of this study was approved by our Institutional Review Board prior to the initiation of the study.

### *Specimen Preparation*

Ten human cadavers (five males and five females) were harvested for this study. All specimens were radiographed to exclude spinal deformity, fracture, or malignancy. One female cadaver was excluded due to the presence of a spinal tumor. Thus, a total of 45 formalin‐fixed lumbar vertebrae (L1–L5) from nine cadavers (five males and four females; mean age: 62.67 years; range: 45–73; standard deviation: 9.37) were tested. The bone mineral density (BMD) was measured in all specimens. Subsequently, all skin, fascia, muscles, ligaments, and capsules were removed, leaving only the intact vertebra which was stored at room temperature until testing.

### *Implantation Procedure*

Both pedicle screws were implanted following the standard protocol for posterior lumbar vertebra implantation. The depth of insertion was consistent and all screws were covered with a sleeve for pull‐out testing prior to implantation. For each vertebra, a traditional pedicle screw was implanted on one side, while a micro‐dynamic pedicle screw was implanted on the other side. All implantations were performed by one surgeon.

### *Test Groups*

The vertebrae were randomly assigned to undergo instant pull‐out testing and 5000‐cyclic and 10,000‐cyclic fatigue loading testing as follows:

- Traditional group and micro‐dynamic group underwent instant pull‐out testing: 15 vertebrae underwent instant pullout testing after inserting traditional pedicle screw on one side and a micro‐dynamic pedicle screw on the other side.
- Traditional group and micro‐dynamic group underwent pull‐out testing after 5000‐cyclic fatigue: 15 vertebrae were inserted with traditional pedicle screw on one side and a micro‐dynamic pedicle screw on the other side, and they then underwent a pull‐out test after 5000‐cyclic fatigue loading testing.
- Traditional group and micro‐dynamic group underwent pull‐out testing after 10,000‐cyclic fatigue: 15 vertebrae were inserted with traditional pedicle screw on one side and a micro‐dynamic pedicle screw on the other side, and they then underwent a pull‐out test after 10,000‐cyclic fatigue loading testing and micro‐CT scanning. The micro‐CT scanning was performed after the cyclic fatigue loading test.

### *Axial Pull‐Out Testing*

**Fig. 2:**
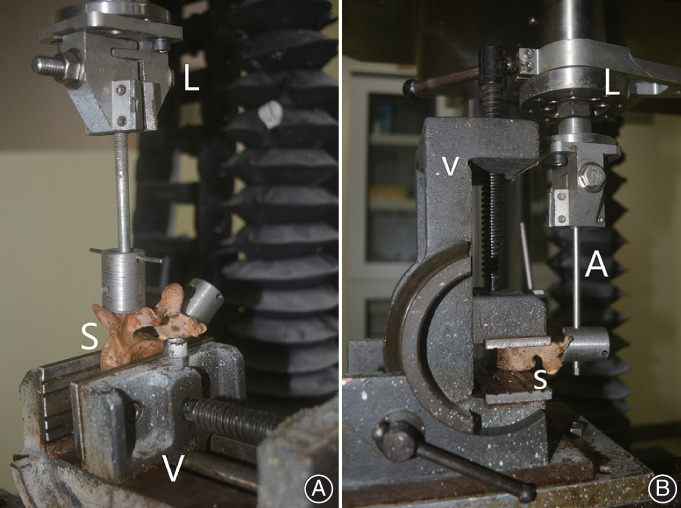
(A) The axial pull‐out: the specimen (S) was gripped using a custom‐designed spine‐testing vice (V) while orienting the vertebrae for the screw to be perpendicular to the ground (parallel to the load cell [L] motion axis); and (B) cycle loading fatigue testing: the specimen (S) was gripped using the custom‐designed spine‐testing vice (V) while orienting the vertebrae for the screw to be parallel to the ground (perpendicular to the load cell [L] motion axis). The hinge‐joint adaptor (A) allowed cycling of the screw.

Traditional group and micro‐dynamic group that underwent instant pull‐out testing were subjected to axial pull‐out testing (Fig. 2A). The specimen was fixed on the BoseAT‐3510 system (Bose, Electro Force Systems Group, Eden Prairie, MN, USA) and adjusted until the longitudinal axis of the screw was in line with the axis of the pull‐out arm of the equipment. Axial pull‐out testing was performed at a rate of 0.5 mm/min until the occurrence of visible screw failure. Our previous research has shown that this rate is appropriate to avoid damage to the vertebrae by preventing sudden pull‐out of the screw^21^. Failure was defined as the point at which there was a precipitous drop in the load–displacement curve. The load–displacement curve was recorded at a frequency of 10 Hz throughout the test. The order of testing for the two sides of each specimen in all groups was random.

### *Cyclic Fatigue Loading Testing*

Traditional group and micro‐dynamic group that underwent pull‐out testing after fatigue loading were subjected to cyclic fatigue loading testing prior to axial pull‐out testing (Fig. 2B). Each specimen was fixed on the BoseAT‐3510 with the axis of the screw perpendicular to the axis of the custom‐designed vice^22^. Two kinds of fatigue testing were conducted, cycled at a rate of 2 Hz for 5000 cycles^23^ and a rate of 2 Hz for 10,000 cycles^24^. The loading pattern applied was displacement control, with the screw displaced 1.5‐mm cephalad and 1.5‐mm caudad from the zero‐load position. The order of testing for the two sides of each specimen in all groups was random, and pull‐out testing was conducted immediately after cyclic fatigue loading for each side.

### *Micro‐CT Image Analysis*

**Fig. 3:**
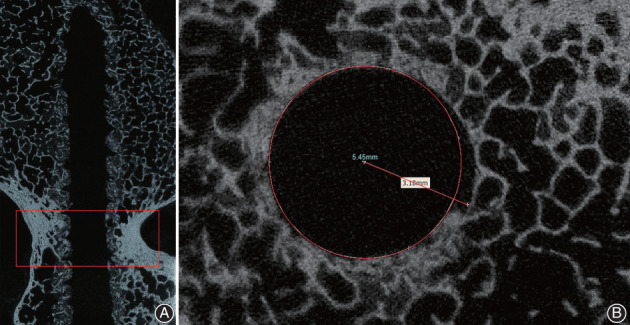
(A) Micro‐CT image of surrounding bone structure of screw insertion area of pedicle zone; and (B) the semidiameter was defined as distance between the thread peek point and the center of the largest circle of insertion cross area.

The samples were scanned with a high‐resolution micro‐CT system (μCT 80, Scanco Medical, AG, Switzerland). The depth of insertion was made consistent, and the scan conditions were as follows: X‐ray voltage 55 kV, current 145 μA, 300 s, continuous non‐stepping rotation, four‐frame averaging, rotation over 360°, and integration time of 300 ms. The surrounding trabecular structure of screws in the vertebral body was damaged due to fatigue loading testing. Therefore, those images were not included in the analysis, and only pedicle zone images were analyzed (Fig. 3A). Pedicle zone was defined as the images cropping between the detected entry point of the pedicle screw and the posterior wall of the vertebral body. A stack of 50–63 cross‐sectional slices was reconstructed, with a slice‐to‐slice distance of 200 μm. The semidiameter was defined as the distance between the thread peak point and the center of the largest circle of the insertion cross area (Fig. 3B). The semidiameter of all slices was measured using the Mimics 14.11 software (Materialize Corp., Leuven, Belgium). The mean value of the semidiameter after the fatigue loading test was used to evaluate screw loosening.

### *Outcome Measurements*

#### *BMD*

Dual‐energy radiograph absorptiometry (DXA, Lunar Prodigy, General Electric, Fairfield, Connecticut) was carried out on all specimens to determine bone mineral density (BMD). Vertebrae with a BMD <0.8 g/cm2 were classified as osteoporotic, whereas those with a BMD >0.8 g/cm2 were classified as healthy^25^.

#### *PPF of Traditional Screw and Micro‐Dynamic Screw*

The peak pull‐out force (PPF) was defined as the peak point of load displacement prior to the occurrence of the precipitous drop.

#### *PPFn of Traditional Screw and Micro‐Dynamic Screw*

In order to determine the performance of the same screw in different vertebrae after various cyclic fatigue loading tests, the normalized PPF (PPFn) values^26^ (i.e., the product PPF of each specimen divided by the appropriate BMD) were compared.

#### *The Mean Value of the Semidiameter on Micro‐CT Image*

The semidiameter was defined as the distance between the thread peak point and the center of the largest circle of the insertion cross area, semidiameter of all insertion cross slices were measured and the mean value of the semidiameter after the fatigue loading test was used to evaluate screw loosening.

### *Statistical Analyses*

Statistical analyses were performed using the SPSS 20.0 software (IBM Corporation, Armonk, NY, USA), and the significance level was defined as *P* < 0.05. Paired *t*‐tests were used to compare paired PPF, BMD, and the mean value of the semidiameter after the fatigue loading test for different screws. One‐way analysis of variance was used to examine the difference of the PPFn of the same screw among the three groups for different cycle conditions and the difference of PPF of traditional pedicle screw and micro‐dynamic pedicle screw for different cycle conditions with and without divided by BMD. Of note, Dunnett's T3 test was used to compare intragroup differences. Lastly, Pearson bivariate correlation was used to investigate the correlation between BMD and PPF.

## Results

### *BMD*

**Fig. 4:**
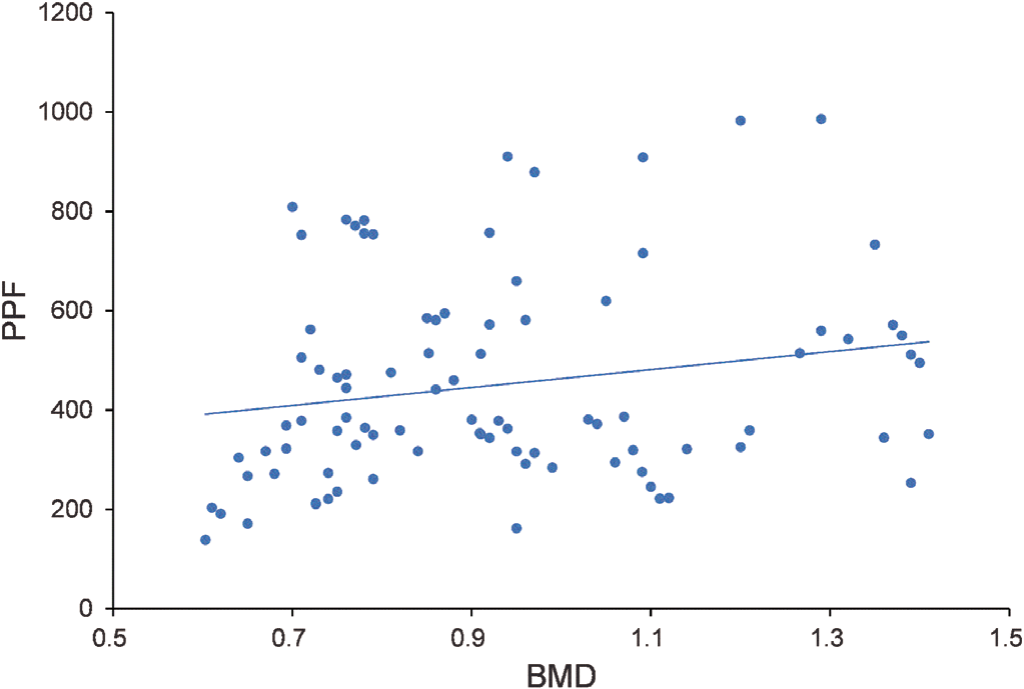
The BMD (g/cm^2^) was weak significant correlation with peak pull‐out force (PPF, N).

The mean BMD, PPF, and PPFn are shown in Table 1. The overall mean BMD of the lumbar vertebrae was 0.89 g/cm^2^ (range: 0.60–1.40; standard deviation: 0.22), and showed a weak significant correlation with PPF (r = 0.252, *P* = 0.024) (Fig. 4).

**TABLE 1:** The mean bone mineral density (BMD), peak pull‐out force (PPF), and normalized PPF (PPFn) for BMD in all groups

Test	Specimen (n)	Mean BMD, g/cm^2^ (SD, range)	Mean PPF, N (SD, range)	Mean PPFn, N/(g/cm^2^) (SD, range)
Instant pull‐out test				
Traditional group	15	0.93 (0.25, 0.73–1.40)	558.41 (218.95, 235.53–908.69)	628.52 (296.86, 244.15–1251.64)
Micro‐dynamic group			534.16 (236.45, 222.94–985.82)	613.34 (350.65, 221.83–1357.88)
Fatigue loading of 5,000 cycles				
Traditional group	15	0.87 (0.11, 0.73–1.01)	471.54 (158.06, 275.12–910.06)	561.12 (243.49, 273.75–1253.53)
Micro‐dynamic group			418.84 (178.20, 210.35–782.80)	500.65 (266.69, 220.69–1078.24)
Fatigue loading of 10,000 cycles				
Traditional group	15	0.89 (0.31, 0.60–1.40)	307.16 (145.71, 138.48–659.43)	304.95 (93.82, 174.29–471.02)
Micro‐dynamic group			413.63 (199.16, 171.21–878.70)	417.03 (146.30, 215.51–627.64)

### *PPF of Traditional Screw and Micro‐Dynamic Screw*

**Fig. 5:**
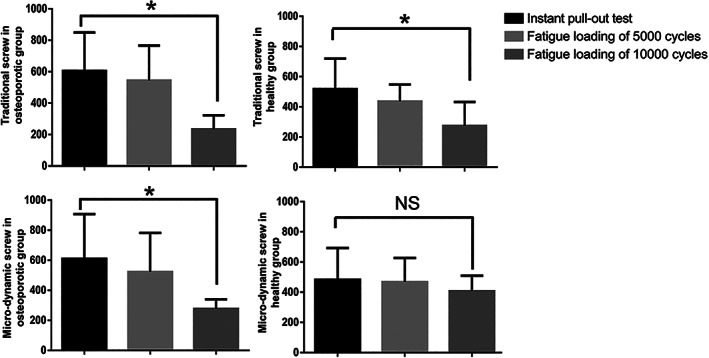
The comparison of the peak pull‐out force (PPF, N) of the traditional screw and micro‐dynamic screw in different groups defied by BMD (osteoporotic and healthy, g/cm^2^). *P < 0.05; NS, no significant difference.

**Fig. 6:**
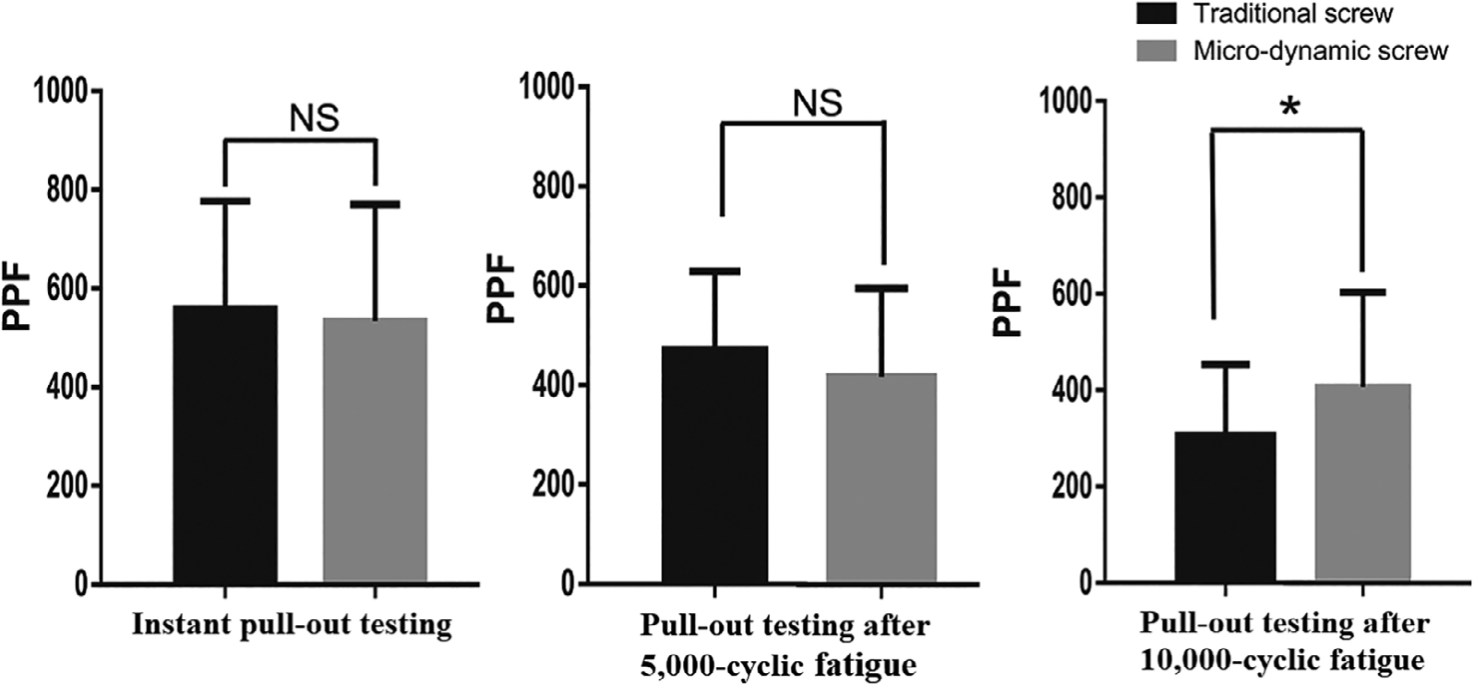
The peak pull‐out force (PPF, N) of the traditional screw and micro‐dynamic screw did not show significant difference in the instant pull‐out test (*P* = 0.485) and fatigue loading for 5000 cycles (*P* = 0.184). The PPF of the micro‐dynamic screw was significantly larger than that of the traditional screw after fatigue loading for 10000 cycles (*P* = 0.005). *P < 0.05; NS, no significant difference.

The PPF of traditional pedicle screw in fatigue loading for 10,000 cycles was 38.64% of that in instant pull‐out test in osteoporotic group (*P* = 0.017) and 66.42% in healthy group (*P* = 0.029); in addition, the PPF of micro‐dynamic pedicle screw in fatigue loading for 10,000 cycles was 45.39% of that in instant pull‐out test in osteoporotic group (*P* = 0.033), but there was no significant difference in healthy group (*P* = 0.853) (Fig. 5). The PPF of the traditional pedicle screw and micro‐dynamic pedicle screw did not show significant differences in the instant pull‐out test (*P* = 0.485) and fatigue loading for 5000 cycles (*P* = 0.184) (Fig. 6). However, the PPF of the micro‐dynamic pedicle screw was significantly larger than that of the traditional pedicle screw after fatigue loading for 10,000 cycles (*P* = 0.005) (Fig. 6).

### *PPFn of Traditional Screw and Micro‐Dynamic Screw*

**Fig. 7:**
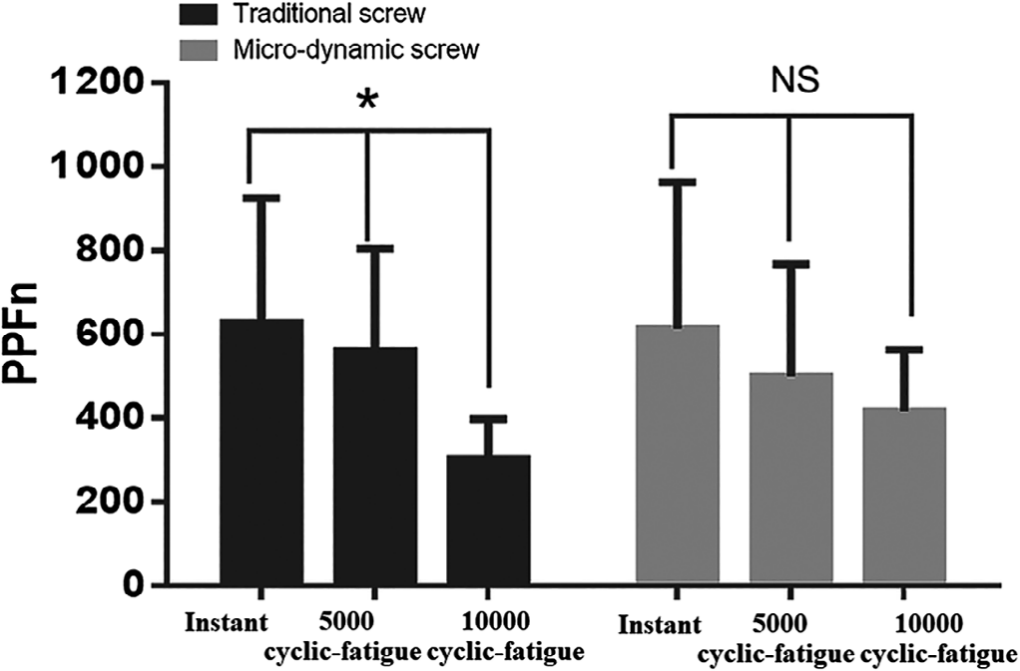
The normalized peak pull‐out force (PPFn, N/(g/cm^2^)) (*P* < 0.001) of the traditional screw was significantly decreased as the number of cycles increased. Meanwhile, the PPFn (*P* = 0.133) of the micro‐dynamic screw remained consistent regardless of the number of cycles. *P < 0.05; NS, no significant difference.

The PPFn of the traditional pedicle screw was significantly decreased as the number of cycles increased (*P* < 0.001) (Fig. 7). Meanwhile, the PPFn of the micro‐dynamic pedicle screw was consistent regardless of the number of cycles (*P* = 0.133) (Fig. 7).

### *The Mean Value of the Semidiameter on Micro‐CT Image*

**Fig. 8:**
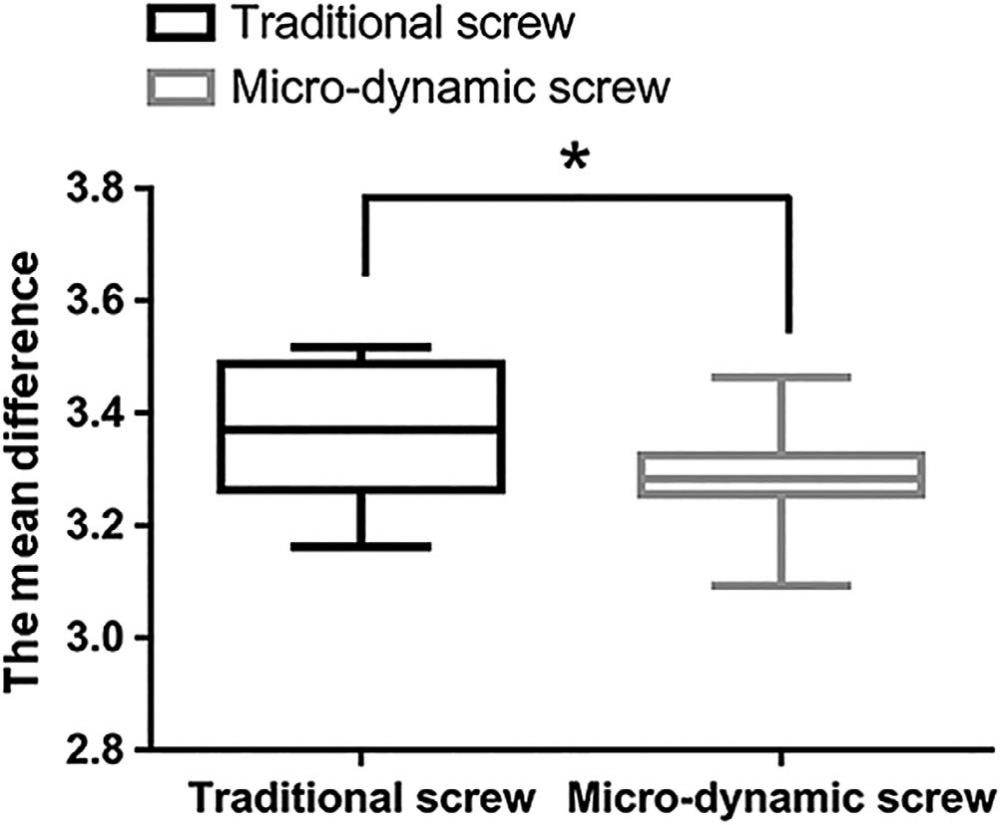
The box plot of mean value of the semidiameter (mm) after the fatigue loading test of traditional screw and micro‐dynamic screw, *P < 0.05.

The mean value of the semidiameter after the fatigue loading test for the novel micro‐dynamic pedicle screw was significantly smaller than that observed for the traditional pedicle screw (*P* = 0.013) (Fig. 8).

## Discussion

In the present study, we investigated the effectiveness of a novel micro‐dynamic pedicle screw for the prevention of loosening between the screw and bone surface using pull‐out testing under instant and cyclic fatigue conditions and micro‐CT scanning. The results indicated that the novel micro‐dynamic pedicle screw provides similar fixation stability with that provided by the traditional pedicle screw for low‐cycle fatigue and osteoporotic group. Moreover, we observed that the micro‐dynamic pedicle screw maintains great resistance even after prolonged cyclic fatigue and in the healthy group. Furthermore, several additional aspects were addressed, such as BMD testing and micro‐CT scanning and the stability of the novel micro‐dynamic pedicle screw.

### *BMD and Micro‐CT Scanning*

Various studies have demonstrated that BMD is a critical factor in determining the pull‐out force^27^, ^28^. However, this study has highlighted the fact that BMD has a weak or no correlation with the pull‐out force^13^. The reason may be that DEXA, a two‐dimensional technique, is unable to capture the three‐dimensional structure and true volumetric BMD (g/cm^3^). Hohn *et al*.^29^ reported that the effectiveness of the standard procedure for determining spine BMD through DEXA scans may be limited in surgical planning due to its inability to assess the variation of BMD within the vertebrae. In addition, Wang *et al*.^30^ demonstrated that the vertebral body contributes on average two‐thirds of the vertebral volume in the lumbar spine and only one‐third of the BMD. They reported a weak correlation between BMD in the posterior elements and the vertebral body (r = 0.34, *P* < 0.0001), further suggesting that DEXA alone is an insufficient predictor of lumbar bone quality for subregions of the vertebrae, such as the pedicle. Thus, we used micro‐CT scanning to investigate screw loosening by comparing the surrounding bone structure of the insertion area. According to Nakashima *et al*.^31^, the surrounding bone structure on micro‐CT images can examine screw loosening after different insertion number. In our study, the mean value of the semidiameter of the micro‐dynamic pedicle screw insertion area on micro‐CT images after the fatigue loading test was significantly smaller than that reported for the traditional pedicle screw, suggesting that the pull‐out force of the traditional pedicle screw significantly decreased in high‐cycle fatigue loading.

### *Stability of the Novel Micro‐Dynamic Pedicle Screw*

Rigid implants prevent stability by controlling the movement between implants and segments. High shear stress and stiffness after rigid fixation increase the load transfer through the implant and bone‐implant interface, leading to damage of the bone and early failure of the interface^32^, ^33^, ^34^. The basic mechanics of the micro‐dynamic pedicle screw reduce the stress concentration between the parts of the implant and the bone‐implant interface, permitting physiological load transfer and appropriate spinal motion. The results of our study show that the resistance of the traditional pedicle screw and micro‐dynamic pedicle screw is not significantly different under both instant pull‐out testing and fatigue loading for 5000 cycles. However, following an increase in the fatigue loading to 10,000 cycles, the micro‐dynamic pedicle screw exhibited stronger resistance. These results suggest that the micro‐dynamic design diffuses the stress concentrated on the bone‐implant interface, delaying the progress of screw loosening and failure. Meanwhile, the micro‐dynamic pedicle screw provides reasonable stress distribution after spinal fusion owing to the micro‐movement design. Moreover, appropriate transfer of stress to adjacent segments after fusion can reduce adjacent segment degeneration. Rienmüller *et al*.^35^ reported a low revision rate in patients undergoing implantation of the dynamic screw system, and reduction in the rates of screw loosening and adjacent segment degeneration. Furthermore, Perez‐Orribo *et al*.^36^ demonstrated that dynamic screw fixation exerts a biomechanically protective effect to the adjacent intact levels and moderately stabilizes the surgical level. Those reports are consistent with our experimental results that dynamic screw can reduce in the rate of screw loosening and delay the loosening process.

This study has several limitations that should be taken into consideration. Firstly, all specimens were formalin‐fixed. Thus, the bone condition was different vs that of fresh bones, which may affect the resistance to the pull‐out force and micro‐CT scanning. Secondly, micro‐CT scanning was only performed on the pedicle zone, while the vertebral body zone was not measured. This may also affect the stability of the pedicle screw.

In conclusion, this study compared the performance of a novel micro‐dynamic pedicle screw vs that of a traditional pedicle screw using pull‐out testing and micro‐CT scanning. The results showed that the novel micro‐dynamic pedicle screw provides similar resistance in low‐cycle fatigue testing and favorable resistance in high‐cycle fatigue testing vs the traditional pedicle screw, and closer combination between the pedicle screw and bone. In addition, the micro‐dynamic pedicle screw provide a better fixation in the healthy group rather than the osteoporotic group, which means fixation for osteoporotic patients requires caution even using micro‐dynamic pedicle screw. The findings of this study might be beneficial in clinic. Besides, high‐quality and scale‐randomized controlled clinical trials are needed to prove findings of our study.
